# Prevalence and genotyping of *Trichomonas* infections in wild birds in central Germany

**DOI:** 10.1371/journal.pone.0200798

**Published:** 2018-08-09

**Authors:** Petra Quillfeldt, Yvonne R. Schumm, Carina Marek, Viktoria Mader, Dominik Fischer, Melanie Marx

**Affiliations:** 1 Department of Animal Ecology & Systematics, Justus Liebig University Giessen, Heinrich-Buff-Ring 26, Giessen, Germany; 2 Clinic for Birds, Reptiles, Amphibians and Fish, Veterinary Faculty, Justus Liebig University Giessen, Frankfurter Strasse 112, Giessen, Germany; Linnaeus University, SWEDEN

## Abstract

Avian trichomonosis is a widespread disease in columbids and other birds, caused by ingestion of the unicellular flagellate *Trichomonas gallinae* which proliferate primarily in the upper respiratory tracts. Studies using genetic analyses have determined some highly pathogenic lineages in birds, but the prevalence and distribution of potentially pathogenic and non-pathogenic *T*. *gallinae* lineages in wild birds is still not well known. We examined 440 oral swab samples of 35 bird species collected between 2015 and 2017 in Hesse, central Germany, for *Trichomonas* spp. infection and for determining the genetic lineages. Of these birds, 152 individuals were caught in the wild and 288 individuals were admitted from the wild to a veterinary clinic. The overall *Trichomonas* spp. prevalence was 35.6%. We observed significant differences between bird orders, with the highest prevalence in owls (58%) and columbids (50%), while other orders had slightly lower prevalences, with 36% in Accipitriformes, 28% in Falconiformes and 28% in Passeriformes. Among 71 successfully sequenced samples, we found 13 different haplotypes, including two previously described common lineages A/B (20 samples) and C/V/N (36 samples). The lineage A/B has been described as pathogenic, causing lesions and mortality in columbids, raptors and finches. This lineage was found in 11 of the 35 species, including columbids (feral pigeon, woodpigeon, stock dove), passerines (greenfinch, chaffinch, blackbird) and raptors (common kestrel, sparrowhawk, red kite, peregrine falcon and common buzzard). One new lineage (R) was found in a sample of a chaffinch. In conclusion, we found that the prevalence of *Trichomonas* spp. infection in wild birds was high overall, and the potentially pathogenic lineage A/B was widespread. Our findings are worrying, as epidemic outbreaks of trichomonosis have already been observed in Germany in several years and can have severe negative effects on bird populations. This disease may add to the multiple pressures that birds face in areas under high land-use intensity.

## Introduction

The flagellate parasite *Trichomonas gallinae* (Rivolta 1878) infects captive and wild birds across the world and can lead to the avian disease called trichomonosis [1]. The first signs typically include white to yellowish oral and pharyngeal lesions. As inflammation and ulceration progress, the lesions may extend to the esophagus, crop and proventriculus and may cause obstipation. Spreading of the pathogen is possible by penetrating the underlying tissues and inner organs such as the liver [2]. Because similar symptoms have been observed in fossils of dinosaurs, trichomonosis is believed to be an ancient pathogen [3].

*Trichomonas* spp. infection is widespread, especially in columbids (e.g. [4]). However, *Trichomonas* spp. infections have also been found in other bird groups such as raptors in Germany [5] and Spain [6] and passerines in Germany [7], the UK [8], USA [9] and Finland [10] and it is thought to play a role in the regulation of wild bird populations [8,11]. The rapid, widespread emergence of finch trichomonosis across Great Britain in 2005 led to the hypothesis that the disease emerged by *T*. *gallinae* jumping from Columbiformes to Passeriformes [12]. The introduction of a novel pathogen into a naive population (i.e. emerging infectious disease), may result in increased morbidity and mortality [13] and is therefore relevant to wildlife conservation. In Germany, only a few studies have reported avian trichomonosis (northern goshawk *Accipiter gentilis* [5], in a single stock dove *Columba oenas* [14]; in greenfinch *Chloris chloris* [7], in 13 urban bird species [15], and columbids: [4]), but only the latter used genetic analyses to characterize the lineages.

Earlier studies [16] found that there are individual differences in disease response and not all infected birds show clinical signs. Presumed reasons include individual host factors (e.g. individual immune status) and differences in virulence of the parasite, which varies among lineages [6,8]. Moreover, some birds showing clinical signs of the disease recover after some days [16]. On the other hand, there are highly pathogenic lineages, which are often harmful to birds and cause lesions in the oropharynx and the liver, and lead to death in almost every case [16]. However, Stabler [17] also demonstrated experimentally that immunisation with a less pathogenic *Trichomonas* spp. lineage is possible. Therefore, it is important to characterize the lineages and to take their different mortality rates into account in order to determine potential effects on a population level.

Here, we study the prevalence and genotypes of *Trichomonas* spp. infections in wild birds in central Germany, including individuals caught in the wild and individuals admitted from the wild to a veterinary clinic. We were interested in the prevalence of *Trichomonas* spp. genotypes in a range of bird species from different orders, and compared the prevalence in wild birds sampled in their breeding and/or migrating habitat versus birds admitted to the veterinary clinic (i.e. previously wild, but injured/diseased birds under veterinary care when oral swab samples were recorded. In particular, we tested the following hypotheses:

1. The prevalence of *Trichomonas* spp. is highest in the order Columbiformes.
2. *Trichomonas* spp. is widespread also among other bird orders.
3. The prevalence is similar in wild birds sampled *in situ* versus wild birds admitted to a veterinary clinic.

## Methods

All sampling was performed in accordance to animal welfare standards, supervised by the Animal welfare officer of the University of Giessen and under permit from the Vogelschutzwarte (Bird Protection Unit).

We examined 440 oral swab samples of 35 bird species collected between 2015 and 2017 in Hesse, central Germany (Table 1). Of these birds, 152 individuals were caught in the wild, either as nestlings at the nest (34 nestlings from nest boxes and natural cavities: 17 stock doves, 15 little owls *Athene noctua* and 2 sparrowhawks *Accipiter nisus*) or during mist-net captures in September 2015 and 2016 in forest and agricultural habitats (118 individuals caught by mist netting). All sampling was performed in accordance to animal welfare standards and under permit. Furthermore, we examined 288 oral swab samples collected during routine clinical examination from previously wild birds admitted to a veterinary clinic. These birds were brought by the public, mostly after being found injured (e.g. after collision with cars or buildings) or showing signs of morbidity such as lethargy and reluctance to flee.

**Table 1:** Prevalence of *Trichomonas* infection in adults of 35 bird species (including nestlings and adults, Ad) with sampling origin in Hesse, Germany.

**Order + Species**		**Age + origin**	**N**	**N** **(AA)**	**Positive** **(PCR)**	**Prevalence (N>3)**	**Sequ.**	***T*.** ***gallinae***	**A/B**	***T*. *tenax***	***T*. *vaginalis***
**Accipitriformes**		**Ad**	**77**	**50**	**18**	**36%**	**11**	**9**	**4**	**0**	**2**
Black kite	*Milvus migrans*	Ad (clinic)	1	-	-	-					
Common buzzard	*Buteo buteo*	Ad (clinic)	41	27	6	22.2%	2	2	1	-	-
Honey buzzard	*Pernis apivorus*	Ad (clinic)	1	1	1	-	1	1	-	-	-
Northern goshawk	*Accipiter gentilis*	Ad (clinic)	14	9	5	55.6%	3	3	-	-	-
Red kite	*Milvus milvus*	Ad (clinic)	6	6	3	50.0%	3	1	1	-	2
Sparrowhawk	*Accipiter nisus*	Nestlings	2	-	-	-					
Sparrowhawk	*Accipiter nisus*	Ad (clinic)	14	7	3	42.9%	2	2	2	-	-
**Anseriformes**	*** ***	** **									
Egyptian goose	*Alopochen aegyptiaca*	Ad (clinic)	1	-	-		1	1	-	-	-
**Columbiformes**	*** ***	** Ad**	**101**	**54**	**27**	**50%**	**34**	**34**	**8**	**0**	**0**
Collared dove	*Streptopelia decaocto*	Ad (clinic)	6	3	2	66.7%	1	1	-	-	-
Feral pigeon	*Columba livia*	Ad (clinic)	32	13	10	76.9%	15	15	5	-	-
Stock dove	*Columba oenas*	Nestlings	17	-	-	-	1	1	1	-	-
Wood pigeon	*Columba palumbus*	Ad (clinic)	63	38	15	39.5%	17	17	3	-	-
**Order + Species**		**Age + origin**	**N**	**N (AA)**	**Positive** **(PCR)**	**Prevalence (N>3)**	**Sequ.**	***T*.** ***gallinae***	**A/B**	***T*. *tenax***	***T*. *vaginalis***
**Falconiformes**	*** ***	** Ad**	**54**	**32**	**9**	**28%**	**5**	**5**	**2**	**0**	**0**
Common kestrel	*Falco tinnunculus*	Ad (clinic)	47	29	7	24.1%	4	4	1	-	-
Eurasian hobby	*Falco subbuteo*	Ad (clinic)	3	1	0						
Peregrine falcon	*Falco peregrinus*	Ad (clinic)	4	2	2		1	1	1	-	-
**Passeriformes**	*** ***	** Ad**	**132**	**123**	**34**	**28%**	**12**	**10**	**5**	**2**	**0**
Black redstart	*Phoenicurus ochruros*	Ad (wild)	2	2	0						
Blackbird	*Turdus merula*	Ad (wild)	29	29	10	34.5%	6	5	2	1	-
Blackcap	*Sylvia atricapilla*	Ad (wild)	1	1	0						
Bullfinch	*Pyrrhula pyrrhula*	Ad (wild)	7	7	1	14.3%	0				
Bullfinch	*Pyrrhula pyrrhula*	Ad (clinic)	1	-	-						
Carrion crow	*Corvus corone*	Ad (clinic)	1	-	-.						
Chaffinch	*Fringilla coelebs*	Ad (wild)	15	15	4	26.7%	3	2	1	1	-
Chaffinch	*Fringilla coelebs*	Ad (clinic)	2	1	0						
Common redstart	*Phoenicurus phoenicurus*	Ad (wild)	3	3	0						
European robin	*Erithacus rubecula*	Ad (wild)	40	40	11	27.5%	1	1	-	-	-
European robin	*Erithacus rubecula*	Ad (clinic)	1	1	1		0				
**Order + Species**		**Age + origin**	**N**	**N (AA)**	**Positive** **(PCR)**	**Prevalence (N>3)**	**Sequ.**	***T*.** ***gallinae***	**A/B**	***T*. *tenax***	***T*. *vaginalis***
**Passeriformes**	*** ***	** **									
Goldfinch	*Carduelis carduelis*	Ad (clinic)	1	1	1						
Great tit	*Parus major*	Ad (wild)	5	5	0	0.0%					
Greenfinch	*Chloris chloris*	Ad (wild)	4	4	1	25.0%	1	1	1	-	-
Greenfinch	*Chloris chloris*	Ad (clinic)	7	1	1	-	1	1	1	-	-
Jackdaw	*Corvus monedula*	Ad (clinic)	1	1	0						
Song thrush	*Turdus philomelos*	Ad (wild)	10	10	4	40.0%	0				
Tree sparrow	*Passer montanus*	Ad (wild)	1	1	0						
Yellowhammer	*Emberiza citrinella*	Ad (wild)	1	1	0						
**Strigiformes**	*** ***	** Ad**	**41**	**19**	**11**	**58%**	**8**	**8**	**5**	**2**	**0**
Barn owl	*Tyto alba*	Ad (clinic)	12	2	2	-	2	2	-	-	-
Boreal owl	*Aegolius funereus*	Ad (clinic)	2	1	0						
Eagle owl	*Bubo bubo*	Ad (clinic)	13	7	4	57.1%	3	3	-	-	-
Little owl	*Athene noctua*	Nestlings	15	-	-						
Little owl	*Athene noctua*	Ad (clinic)	1	1	1						
Long-eared owl	*Asio otus*	Ad (clinic)	6	3	1	33.3%	1	1	-	-	-
Tawny owl	*Strix aluco*	Ad (clinic)	7	5	3	60%	2	2	-	-	-
**Overall total**		** Ad**	**406**	**278**	**99**	**36%**	**71**	**66**	**24**	**4**	**2**

Nestlings were sampled in the wild. A prevalence was calculated based on ammonium acetate-extracted samples (AA), for sample sizes>3. Furthermore, numbers of sequences (Sequ.) are given together with *Trichomonas* species and the numbers of birds infected with the potentially pathogenic *Trichomonas gallinae* lineage A/B.

Oral swabs were taken from the oropharynx with a dry, sterile cotton tip, and swabs were inoculated individually in a *Trichomonas*-selective culture medium (Trichomonas medium REF EB0861C, OXOID Deutschland GmbH, Wesel, Germany). The samples were incubated at 37°C for five to seven days and extracted using an ammonium-acetate (AA) protocol (278 samples from 2014 to 2016 [18]) or a DNAzol protocol (162 samples from 2017, [4]). Although the latter protocol is faster, and has been recommended (Kevin Tyler und Abdulwahed Alrefaei, pers. comm.) and used ([4]), the AA protocol yielded statistically higher prevalences, both when all samples were considered (AA: 36%, DNAzol: 19%, Chi-squared test, χ^2^ = 13.5, d.f. = 1, p < 0.001) or only the samples from the clinic birds (AA: 42%, DNAzol: 23%, Chi-squared test, χ^2^ = 11.8, d.f. = 1, p < 0.001). All wild caught samples of adults were extracted using the PBS protocol, while the birds from the avian clinic were distributed between the methods (160 PBS, 128 DNAzol), and all nestlings were extracted using the DNAzol method. Because the apparent prevalence was lower using DNAzol, the prevalences were calculated only for the AA-extracted samples, but genotyped DNAzol-extracted samples are included in phylogenetic analyses.

The DNA was re-suspended in 30 μl of ddH_2_O. The DNA concentration of all samples was measured using a ThermoScientific Nanodrop 2000 micro-volume UV-VIS spectrometer. Samples with a DNA-concentration above 70ng/μl were diluted to a final concentration of 20ng/μl. The AA protocol yielded higher Nanodrop DNA concentrations than the DNAzol protocol (AA: 76.7 ng/μl, DNAzol: 17.7 ng/μl, Welch Two Sample t-test, t = 2.6, d.f. = 67.3, p = 0.016).

For the PCR detection of *Trichomonas* spp. infections, we amplified the highly conserved ITS1-5.8S-ITS2 ribosomal region of the *T*. *gallinae* genome (Grabensteiner et al. 2010) with the primers TFR1 (5′-TGC TTC AGT TCA GCG GGT CTT CC-3′) and TFR2 (5′-CGG TAG GTG AAC CTG CCG TTG G-3′) [19]. We used 20 μl reaction volume per sample, including 10 μl DreamTaq PCR Mastermix (2x, Thermo Scientific, Germany), 2.5 μl of forward and reverse primers (both 20 μM), 6 μl ddH2O and 3 μl of template DNA.

PCR reactions were conducted on a Biometra TPersonal Thermocycler (Biometra, Göttingen, Germany) with the following cycling conditions: polymerase activation at 95°C for 15 min, followed by 35 cycles with a denaturation at 94°C for 30 s, annealing at 60°C for 90 s and extension at 72°C for 60 s. Final extension was set to 72°C for 10 min. All PCR reactions were run with a negative and positive control.

We used capillary electrophoresis (QIAxcel Advanced, Qiagen, Switzerland) to visualise PCR products. Positive samples were purified with innuPREP PCRpure Kit (Analytik Jena, Germany). In total, 130 PCR products were sent for bi-directional Sanger sequencing to SEQLAB (Sequence Laboratories Göttingen, Germany).

Forward and reverse sequences were assembled and trimmed with CLC Main Workbench 7.6.1 (CLC bio, Qiagen). Sequences with any uncertain positions were repeated and if they did not improve, were not included in the alignment. By checking the trace data, we concluded that for most omitted sequences technical issues such as lows yields of amplicons led to poor quality sequences. However, in five sequences (from oral swabs of one feral pigeon, one Woodpigeon, two common buzzards and one goshawk) double peaks were present in the chromatograms, which might indicate mixed infections.

In total, we retained 71 consensus sequences. All newly generated sequences were submitted to GenBank (accession numbers MH459299-MH459369).

We checked every sequence for its closest GenBank match in NCBI Blast [20] and downloaded these as reference sequences (see S1 Table for an overview about the percentage of identity to the closest GenBank match). In addition, we used one reference sequence of each *Trichomonas* lineage described in [4] (GenBank accession numbers: lineage A/B: EU881911; lineage II: HM579936; lineage III: KC529665; lineage C/V/N: KF993679; lineage P: KF993705; lineage O: KX459442.1; lineage Q: KX459510). *Tritrichomonas foetus* (GenBank accession number DQ243911.1; Duboucher et al. 2006) was used as outgroup for phylogenetic analysis following previous studies [4, 21].

We aligned all sequences using BioEdit [22], and calculated a Median Joining Network in PopART [23, 24] without reference sequences. A Bayesian phylogenetic tree was generated with BEAST v1.8.4 [25]. The alignment included 71 sample sequences plus 18 reference sequences and an outgroup and had a total length of 307 base pairs (bp). A Tamura 3 parameter substitution model with Gamma distribution was determined as best suitable nucleotide substitution model for our alignment in jModelTest 2.1.7 [26] and MEGA 6.0 [27] using Bayesian Information Criterion scores. Priors were defined using the interface BEAUTi v1.8.4 [28], including strict clock as clock type and a Yule speciation process [29]. Markov Chain Monte Carlo (MCMC) simulations were run with the following settings: ngen = 25,000,000; sample-freq = 1,000; burn-in 10%). Convergence of parameters and their effective sample size (ESS > 200) were confirmed in Tracer v1.6 [30]. A maximum clade credibility tree was computed with TreeAnnotator v1.8.4 [31] and visualised in FigTree v1.4.3 [32].

Statistical analyses of differences in prevalences were carried out using Chi-squared tests in R 3.2.4. [33].

## Results

### Prevalences

The overall prevalence of *Trichomonas spp*. was 35.6% for all AA-extracted birds (99 of 278 individuals; Table 1). There were significant differences between adult individuals from the five different bird orders (Chi-squared test, χ^2^ = 13.6, df = 4, p = 0.010). The highest prevalences were found in adult Strigiformes (58%, N = 19) and Columbiformes (50%, N = 54), while the other orders had slightly lower prevalences, with 36% in adult Accipitriformes (N = 50), 28% of adult Falconiformes (N = 32) and 28% of Passeriformes (N = 123). On a species level, feral pigeons had the highest prevalence (77%, Table 1), but in some species with small sample sizes all samples were positive (honey buzzard N = 1, peregrine falcon N = 2, goldfinch N = 1, little owl N = 1).

Birds admitted to the clinic had a higher prevalence, with 32 of 118 birds (27%) caught in situ positive, compared to 67 of 160 birds (42%) of clinic birds (Chi-squared test, χ^2^ = 6.4, df = 1, p = 0.011).

Nestlings were the least affected group (none of 15 little owls, one of 17 stock doves and one of two sparrowhawks), but as none of these had been AA-extracted, this may include some false negatives. One Egyptian goose *Alopochen aegyptiaca* was also tested positive after DNAzol-extraction.

### Genotyping

Among 71 successfully sequenced samples (S1 Table), we found 13 different haplotypes, including two previously described common lineages A/B (20 samples) and C/V/N (36 samples). The lineage A/B was found in 11 of the 36 bird species (Table 1), including columbids (feral pigeon, woodpigeon, stock dove), passerines (greenfinch, chaffinch *Fringilla coelebs*, blackbird) and raptors (common kestrel *Falco tinnunculus*, sparrowhawk, red kite *Milvus milvus*, peregrine falcon *Falco peregrinus* and common buzzard *Buteo buteo*).

**Fig 1:**
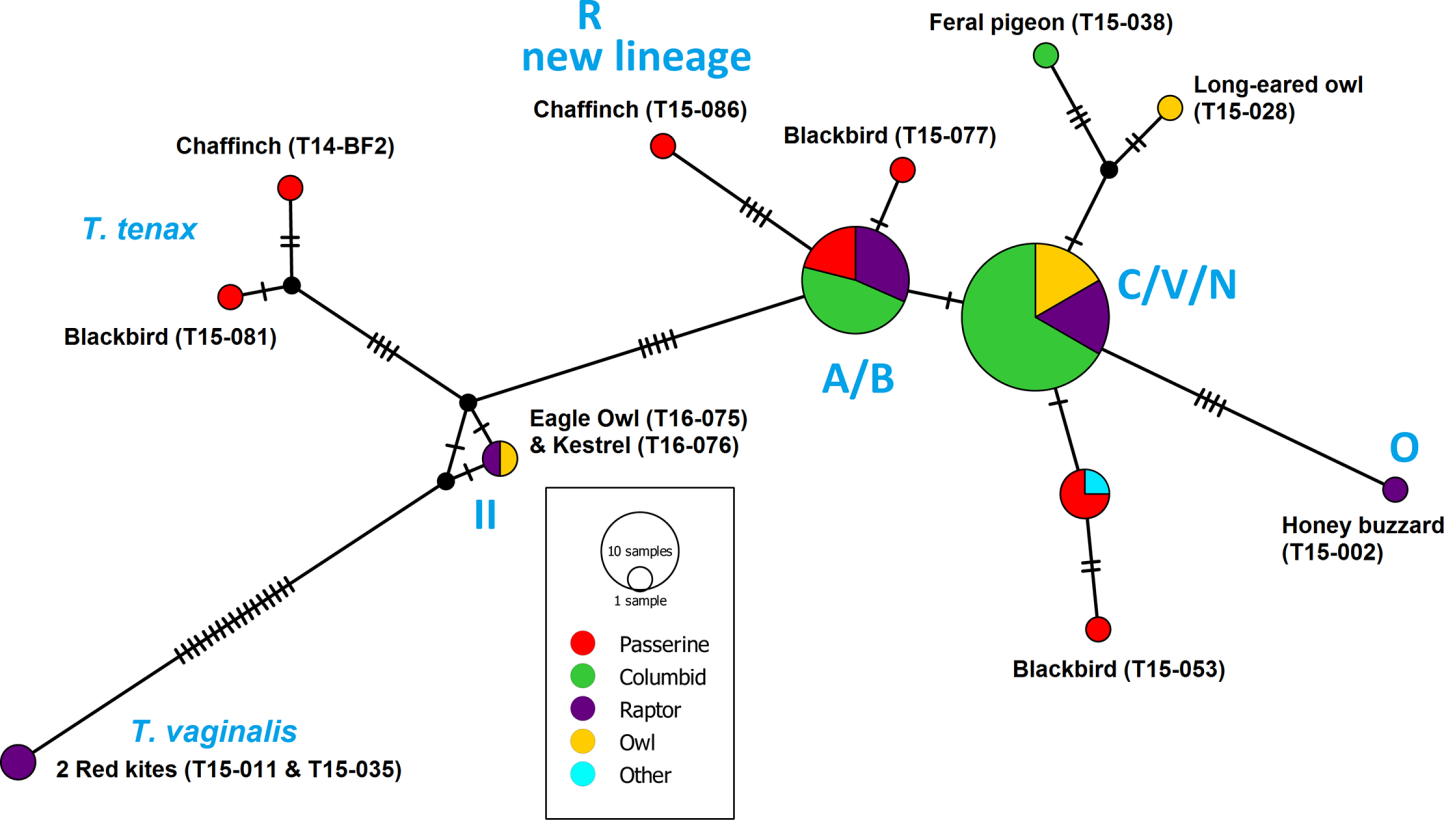
Median-joining haplotype network of the ITS1/5.8S/ITS2 ribosomal region of 71 *Trichomonas* sequences from samples of birds. The size of the colour coded circles is proportional to haplotype frequency. The hatch marks on the lines represent mutational steps.

**Fig 2:**
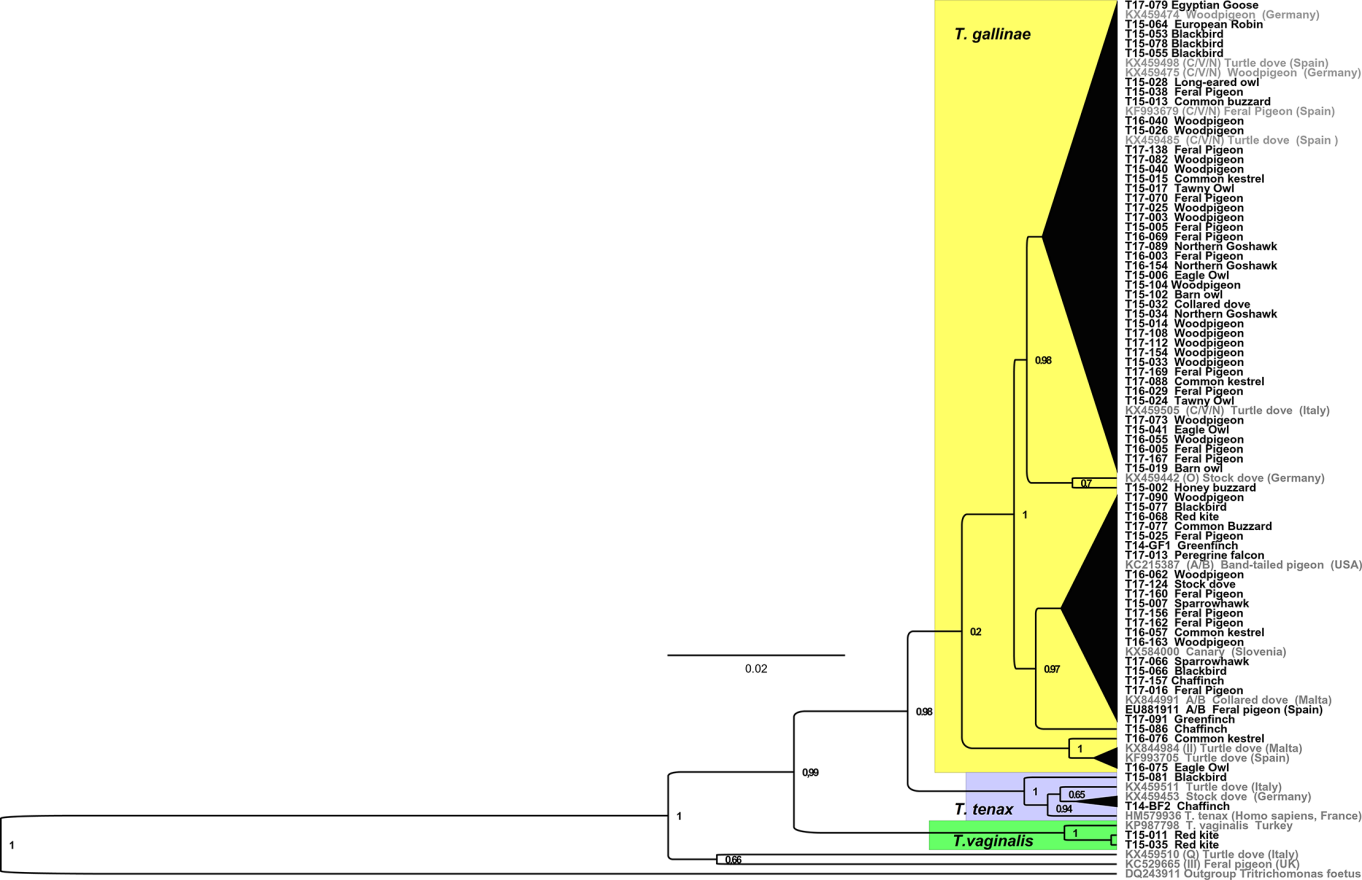
Maximum Clade Credibility Tree of the ITS1/5.8S/ITS2 ribosomal region of 71 *Trichomonas* sequences from samples of birds and reference sequences, using the Maximum Likelihood method in BEAST (Bayesian Evolutionary Analysis by Sampling Trees). Reference sequences (grey) are labelled with GenBank accession number, lineage, host species and sampling site. Sequences sampled in the present study are marked black. The posterior values are given at the nodal points. Branches with posterior value≤ 0.5 were pooled.

The lineage A/B was absent in owls (Table 2) and overall, lineage C/V/N was the most prevalent. One sample of a chaffinch (sample T15-086) could not be assigned to a known lineage and is a new lineage (R, Figs 1 and 2).

**Table 2:** *Trichomonas* species and lineages in bird orders observed in Hesse, Germany.

	A/B	C/V/N	II	O	R	*Trichomonas tenax*	*Trichomonas vaginalis*
Accipitriformes (N = 11)	4	4	-	1			2
Anseriformes (N = 1)	-	1	-	-			
Columbiformes (N = 34)	9	25	-	-			
Falconiformes (N = 5)	2	2	1	-			
Passeriformes (N = 12)	5	4	-	-	1	2	
Strigiformes (N = 8)	-	7	1	-			
All sequences (N = 71)	20	43	2	1	1	2	2

The numbers of consensus sequences per *Trichomonas species* and, in the case of *Trichomonas gallinae*, lineages (A/B, C/V/N, II, O and the new lineage R) are listed.

The haplotypes belonged to three Trichomonas species, *T*. *gallinae*, *T*. *tenax* and *T*. *vaginalis* (Table 1, Fig 2).

## Discussion

Trichomonosis is regarded as an emerging infectious disease in wild birds in Europe [12], and in the present study, we confirmed the widespread prevalence of this pathogen in several bird orders beyond Columbiformes in Germany, including the potentially pathogenic lineage A/B.

### Prevalence

We observed a high prevalence of *Trichomonas* spp. infections in wild birds in central Germany. As expected from the literature and previous studies [4], the prevalence was highest in the order Columbiformes, with nearly half of the birds tested positive. However, *Trichomonas spp*. was also widespread among other bird orders, with a prevalence of 28–36% observed in Passeriformes, Strigiformes and diurnal raptors (Accipitriformes/Falconiformes, Table 1).

The prevalence was higher in wild birds admitted to a veterinary clinic than in wild birds sampled *in situ*. Typically, these birds are found by the public, mostly being injured in traffic or suffering from trauma or other diseases of different geneses. The present data suggest however, that trichmonosis may have contributed to the probability of a bird being injured or found weak. Although trichmonosis is often subclinical and can be kept in check by the immune systems of the birds [16, 17], sublethal effects of parasites may include a decreased breeding success through increased use of resources to keep up immune responses [34] and thus, trichomonosis may predispose the birds to the causes of admission to the clinic. However, the distribution of bird orders in the wild bird and clinic samples differed, and thus, more work is needed to confirm this difference within species of birds. In some species with small sample sizes all samples were positive, and therefore, these species may require particular attention in future research.

Besides wild Columbiformes [4], finches (Passeriformes: Fringillidae) seem to be particularly prone to mortality by trichomonosis, which was the most probable cause for massive declines in breeding populations of greenfinches in the UK [8] and in Scandinavia [10]. In Germany, mortality events have also been recorded, for example an estimated 70.000 to 80.000 greenfinches died in 2009 [35] and Trichomonosis was diagnosed [7]. In the present study, we obtained only small sample sizes of finches, but all four investigated species (greenfinch, goldfinch *Carduelis carduelis*, bullfinch *Pyrrhula pyrrhula* and chaffinch) were found positive for *Trichomonas* spp. Moreover, two greenfinches and one chaffinch were infected with the potentially pathogenic line A/B. Therefore, trichomonosis may also explain decreasing trends of greenfinches recorded in the NABU (Nature and Biodiversity Conservation Union, Germany) citizen science garden counts in Hesse (S1 Fig): Numbers of observed greenfinches decreased in spring from 1.5 to 1.1 individuals per garden over 12 years and even more dramatically in winter from 2.4 to 1.1 individuals per garden over 7 years.

Other stressors may influence the mortality caused by parasites such as *Trichomonas* spp. For example, wild-caught greenfinches in Tartu, Estonia, that died from trichomonosis had a fivefold higher occurrence of fault bars on tail feathers than survivors [36]. Fault bars may be markers of low food availability and stress experienced during feather growth. Therefore, their presence in birds which died from trichomonosis may indicate that these birds had a disturbed immune systems or disturbed organ function (especially liver and kidney) or were impaired in foraging. Anthropogenic pressures such as changes in land use and pollution may also contribute to increasing the mortality rates of infected birds.

Raptors have also been reported as hosts of *Trichomonas* spp. before, in particular in urban areas where transmission may be facilitated by an increased consumption of urban Columbiformes (e.g. [6, 37]). This may also explain why small raptors like kestrels or little owls that do not prey on columbids had lower prevalence.

### Genotyping

The prevalence of *Trichomonas* genotypes varied in different bird orders, although our sample size is too low to be completely conclusive.

The most common *T*. *gallinae* lineage C/V/N was present in all bird orders analysed in this study. This lineage is considered less pathogenic, and an infection with this lineage may even help in building up immunity [17]. According to the haplotype network, the lineage C/V/N consists of several haplotypes, with up to four mutations distinguishing the rarer haplotypes such as the feral pigeon sample (T15-038) from the most common C/V/N haplotype (Fig 1). Some nomenclatures such as the MalAvi database for malaria-like avian haemoparasites [38] define a single mutation, i.e. every haplotype, as a separate lineage. A database for *Trichomonas* lineages similar to the MalAvi database would be useful that unifies the different nomenclatures (e.g. summarised by [4]).

The *Trichomonas gallinae* lineage A/B is considered to be very pathogenic, as indicated by the dramatic decline in the greenfinch population in the United Kingdom in 2007 [8]. The high mortality in greenfinches and chaffinches was attributed to this lineage (A1 according to [39]). This lineage has also been found in raptors [37] and Columbiformes [4, 39]. Sansano-Maestre et al. [6] found that all birds (columbids and raptors) with macroscopic lesions carried this strain. In the present study, two out of eleven greenfinches were tested positive for *T*. *gallinae* and in both the lineage A/B was detected. Overall, this line could be assigned unambiguously to samples of 11 of the 35 species, including columbids (feral pigeon, woodpigeon, stock dove), passerines (greenfinch, chaffinch, blackbird) and raptors (common kestrel, sparrowhawk, red kite, peregrine falcon and common buzzard).

However, the lineage A/B has been found in dead Passeriformes [40], whereas there are only few studies on birds that did not have clinical signs or studies on the course of infection in songbirds. Furthermore, it is unclear whether this lineage has the same virulence in all host species and if some infected individuals can recover from this lineage and develop immunity [17].

Concerning the rarer lineages according to previous surveys (e.g. [4]), we found one honey buzzard infected with lineage O (Fig 1), and one eagle owl and one kestrel infected with lineage II. These findings indicate that these and other more rarely described lineages (e.g. P, Q, [4]) are indeed also rare in non-columbid birds. One sample of a chaffinch (sample T15-086) could not be assigned to a known lineage and is therefore here identified as a new lineage (R).

Two samples of passerines (one chaffinch and one blackbird) were assigned to *Trichomonas tenax*. This species has already been found in other studies in birds, and formed a group together with a *T*. *tenax* pathogen found in humans [19, 41], indicating a close relationship between *T*. *gallinae* and *T*. *tenax* [41].

Finally, two samples of red kites were assigned to *Trichomonas vaginalis*, a pathogen of humans causing human trichomonosis. This species has previously been registered in columbids in the US [42] and in a bearded vulture *Gypaetus barbatus* in the Czech Republic [41].

In summary, we found pathogens of three *Trichomonas* species, *T*. *gallinae*, *T*. *tenax* and *T*. *vaginalis* in the swab samples from a range of bird species in Germany. The potentially pathogenic lineage A/B was widespread. These findings are worrying, because epidemic outbreaks of trichomonosis, which have already been observed in Germany in several years, can have severe negative effects on populations. This disease may add to the multiple pressures that bird populations face in densely populated regions.

## Supporting information

S1 Table *Trichomonas* samples (N = 71) with their closest GenBank match for ITS1/5.8S/ITS2 region, maximum identity and query coverage in % as well as the *Trichomonas* species of the GenBank match, lineage of *Trichomonas gallinae*, host and country in which the reference was found. (PDF)

S1 Fig Decline in greenfinch numbers in Hesse, Germany, according to the spring (https://www.nabu.de/tiere-und-pflanzen/aktionen-und-projekte/stunde-der-gartenvoegel/index.html) and winter garden bird counts (https://www.nabu.de/tiere-und-pflanzen/aktionen-und-projekte/stunde-der-wintervoegel/index.html) of the NABU (Nature and Biodiversity Conservation Union, Germany). (PDF)

## References

[1] Forrester DJ, Foster GW. Trichomonosis In: Atkinson CT, Thompson NJ, Hunter DB,editors. Parasitic diseases of wild birds. Ames, Iowa: Wiley-Blackwell; 2008.

[2] Stabler RM. *Trichomonas gallinae*: A Review. Experim Parasitol. 1954; 3:368–402. 10.1016/0014-4894(54)90035-1 13183096

[3] Wolff ED, Salisbury SW, Horner JR, Varricchio DJ. Common avian infection plagued the tyrant dinosaurs. PLoS One. 2009; 4: e7288 10.1371/journal.pone.0007288 19789646 PMC2748709

[4] Marx M, Reiner G, Willems H, Rocha G, Hillerich K, Masello JF, et al High prevalence of *Trichomonas gallinae* in wild columbids across western and southern Europe. Parasites & Vectors. 2017; 10: 242. 28521843 10.1186/s13071-017-2170-0 PMC5437606

[5] Krone O, Altenkamp R, Kenntner N. Prevalence of *Trichomonas gallinae* in northern goshawks from the Berlin area of northeastern Germany.J Wildl Dis. 2005; 41: 304–309. 10.7589/0090-3558-41.2.304 16107664

[6] Sansano-Maestre J, Garijo-Toledo MM& Gómez-Muñoz MT. Prevalence and genotyping of *Trichomonas gallinae* in pigeons and birds of prey. Avian Pathol. 2009; 38: 201–207. 10.1080/03079450902912135 19468936

[7] Peters M, Kilwinski J, Reckling D, Henning K. Epidemic mortality in greenfinches at feeder stations caused by *Trichomonas gallinae*—a recent problem in Northern Germany. Kleintierpraxis. 2009; 54:433–438.

[8] Robinson RA, Lawson B, Toms MP, Peck KM, Kirkwood JK, Chantrey J. Emerging infectious disease leads to rapid population declines of common British birds. PLoS One. 2010:5. 10.1371/journal.pone.0012215 PMC2923595 20805869

[9] Anderson NL, Grahn RA, Van Hoosear K, BonDurant RH. Studies of trichomonad protozoa in free ranging songbirds: prevalence of *Trichomonas gallinae* in house finches (*Carpodacus mexicanus*) and corvids and a novel trichomonad in mockingbirds (*Mimus polyglottos*). Veterin Parasitol. 2009; 161: 178–186. 10.1016/j.vetpar.2009.01.023 19278788

[10] Lehikoinen A, Lehikoinen E, Valkama J, Väisänen RA, Isomursu M. Impacts of trichomonosis epidemics on Greenfinch *Chloris chloris* and Chaffinch *Fringilla coelebs* populations in Finland. Ibis. 2013; 155: 357–366.

[11] Amin A, Bilic I, Liebhart D, Hess M. Trichomonads in birds—a review. Parasitol. 2014; 141: 733–47. 10.1017/S0031182013002096 24476968

[12] Lawson B, Robinson RA, Colvile KM, Peck KM, Chantrey J, Pennycott TW, et al The emergence and spread of finch trichomonosis in the British Isles. Philos Trans R Soc Lond B Biol Sci. 2012; 367: 2852–63. 10.1098/rstb.2012.0130 22966140 PMC3427565

[13] Daszak P, Cunningham AA & Hyatt AD. Emerging infectious diseases of wildlife: threats to biodiversityand human health. Science. 2000; 287: 443–449. 10642539 10.1126/science.287.5452.443

[14] Hegemann A, Hegemann ED, Krone O. Trichomonosis in a free-living stock dove (*Columba oenas*). European J Wildl Res. 2007; 53: 235–237.

[15] Stenkat J, Krautwald-Junghanns ME, Schmidt V. Causes of morbidity and mortality in free-living birds in an urban environment in Germany. Ecohealth. 2013; 10: 352–365. 10.1007/s10393-013-0868-9 24136384

[16] Stabler RM. Variations in virulence of strains of *Trichomonas gallinae* in Pigeons. J Parasitol. 1948; 34: 147–149. 18856299

[17] Stabler RM. Protection in pigeons against virulent *Trichomonas gallinae* acquired by infection with milder strains. J Parasitol. 1948; 34: 150–153. 18856300

[18] Megía-Palma R, Martínez J, Merino S. Phylogenetic analysis based on 18S rRNA gene sequences of *Schellackia* parasites (Apicomplexa: Lankesterellidae) reveals their close relationship to the genus *Eimeria*. Parasitology. 2013; 140:1149–57. 10.1017/S0031182013000553 23731491

[19] Felleisen RS. Comparative sequence analysis of 5.8S rRNA genes and internal transcribed spacer (ITS) regions of trichomonadid protozoa. Parasitol. 1997; 115:111–119. 10.1017/s0031182097001212 10190167

[20] Altschul SF, Madden TL, Schäffer AA, Zhang J, Zhang Z, Miller W, Lipman DJ. Gapped BLAST and PSI-BLAST: A new generation of protein database search programs. Nucleic Acids Res. 1997; 25: 3389–3402. 9254694 10.1093/nar/25.17.3389 PMC146917

[21] Lennon RJ, Dunn JC, Stockdale JE, Goodman SJ, Morris AJ & Hamer KC. Trichomonad parasite infection in four species of Columbidae in the UK. Parasitol. 2013; 140: 1368–1376. 10.1017/S0031182013000887 23866933

[22] Hall T. BioEdit Biological sequence alignment editor for Win95/98/NT/2K/XP/7. 1997; http://www.mbio.ncsu.edu/BioEdit/bioedit.html

[23] Bandelt H, Forster P, Röhl A. Median-joining networks for inferring intraspecific phylogenies. Mol Biol Evol. 1999; 16: 37–48. 10.1093/oxfordjournals.molbev.a026036 10331250

[24] Leigh JW, Bryant D. PopART: Full-feature software for haplotype network construction. Methods Ecol Evol. 2015; 6:1110–1116.

[25] Drummond AJ, Rambaut A, Suchard MA. BEAST: Bayesian evolutionary analysis sampling trees v1.8.4. 2002. 10.1186/1471-2148-7-214 PMC2247476 17996036

[26] Darriba D, Taboada GL, Doallo R, Posada D. jModelTest 2: more models, new heuristics and parallel computing. Nature Methods. 2012; 9: 772. 10.1038/nmeth.2109 PMC4594756 22847109

[27] Tamura K, Stecher G, Peterson D, Filipski A, Kumar S. MEGA6: Molecular Evolutionary Genetics Analysis Version 6.0. Mol Biol Evol. 2013; 30: 2725–2729. 10.1093/molbev/mst197 24132122 PMC3840312

[28] Drummond AJ, Rambaut A, Suchard MA, Xie W. BEAUTi: Bayesian Evolutionary Analysis Utility Version v 1.8.4. 2016.

[29] Gernhard T. Yule Processs. J Theor Biol. 2008; 253: 769–778. 10.1016/j.jtbi.2008.04.005 18538793

[30] Rambaut A, Suchard MA, Xie W, Drummond AJ, Tracer v1.6.0. 2013.

[31] Drummond AJ, Rambaut A. MCMC Output analysis. TreeAnnotator v1.8.4. 2016

[32] Rambaut, A. FigTree, a graphical viewer of phylogenetic trees. 2012; http://tree.bio.ed.ac.uk/software/figtree

[33] R Core Team. R: A Language and Environment for Statistical Computing. R Foundation for Statistical Computing Vienna, Austria 2016; http://www.Rproject.org.

[34] Graham AL, Allen JE, Read AF. Evolutionary causes and consequences of immunopathology. Ann Rev Ecol Evol Syst. 2005; 36: 373–397.

[35] NABU Schleswig-Holstein. https://schleswig-holstein.nabu.de/tiere-und-pflanzen/voegel/singvögel/finken/12602.html. 2010; revised 1 March 2018

[36] Männiste M, Hõrak P. Emerging infectious disease selects for darker plumage coloration in greenfinches. Frontiers Ecol Evol. 2014; 2: 4.

[37] Boal CW, Mannan RW and Hudelson KS. Trichomoniasis in Cooper's hawks from Arizona. J Wildl Dis. 1998; 34: 590–593. 10.7589/0090-3558-34.3.590 9706569

[38] Bensch S, Hellgren O, Pérez-Tris J. MalAvi: a public database of malaria parasites and related haemosporidians in avian hosts based on mitochondrial cytochrome b lineages. Mol Ecol Res. 2009; 9: 1353–1358. 10.1111/j.1755-0998.2009.02692.x 21564906

[39] Chi JF, Lawson B, Durrant C, Beckmann K, John S, Alrefaei AF et al The finch epidemic strain of *Trichomonas gallinae* is predominant in British non-passerines. Parasitol. 2013; 140, 1234–1245. 10.1017/S0031182013000930 23920088

[40] Lawson B, Cunningham AA, Chantrey J, Hughes LA, John SK, Bunbury N et al A clonal strain of *Trichomonas gallinae* is the aetiologic agent of an emerging avian epidemic disease. Infection, Genetics Evol. 2011; 11: 1638–1645. 10.1016/j.meegid.2011.06.007 21712099

[41] Grabensteiner E, Bilic I, Kolbe T, Hess M. Molecular analysis of clonal trichomonad isolates indicate the existence of heterogenic species present in different birds and within the same host. Vet Parasitol. 2010; 172: 53–64. 10.1016/j.vetpar.2010.04.015 20471174

[42] Gerhold RW, Yabsley MJ, Smith AJ, Ostergaard E, Mannan W, Cann JD et al Molecular characterization of the *Trichomonas gallinae* morphologic complex in the United States. J Parasitol. 2008; 94: 1335–1341. 10.1645/GE-1585.1 18576862

