# Supplementary material for: Prevalence and genotyping of Trichomonas infections in wild birds in central Germany
Source: PLoS One. 2018 Aug 9;13(8):e0200798. doi: 10.1371/journal.pone.0200798 (PMC6084888; doi:10.1371/journal.pone.0200798)
Supplement: S1 Table — (PDF) [file pone.0200798.s001.pdf]

Prevalence and genotyping of Trichomonas infections in wild birds in central Germany

Petra Quillfeldt, Yvonne R. Schumm, Carina Marek, Viktoria Mader, Dominik Fischer and Melanie Marx

Supplementary material

S1 Table: Trichomonas samples (N=71) with their closest GenBank match for ITS1/5.8S/ITS2 region, maximum identity and query coverage in % as well as the *Trichomonas* species of the GenBank match, lineage of *Trichomonas gallinae*, host and country in which the reference was found.

Order+ Host species	Sample ID	*Trichomonas species*	Lineage	Accession number	Identity	Query cover	Host species	country
**Accipitriformes**								
Common buzzard	T15-013	*T. gallinae*	C/V/N	KX459475	100%	99%	Woodpigeon	Germany
Common buzzard	T17-077	*T. gallinae*	A/B	KX844991	100%	100%	Collared dove	Malta
Honey buzzard	T15-002	*T. gallinae*	O	KX459498	**98%**	99%	Turtle dove	Spain
Northern Goshawk	T15-034	*T. gallinae*	C/V/N	KX459497	100%	99%	Turtle dove	Spain
Northern Goshawk	T16-154	*T. gallinae*	C/V/N	KX459474	100%	100%	Woodpigeon	Germany
Northern Goshawk	T17-089	*T. gallinae*	C/V/N	KX459476	100%	100%	Woodpigeon	Germany
Red kite	T15-011	*T. vaginalis*	-	KP987798	**97%**	99%	unknown	Turkey
Red kite	T15-035	*T. vaginalis*	-	KP987798	**97%**	99%	unknown	Turkey
Red kite	T16-068	*T. gallinae*	A/B	KX844991	100%	100%	Collared dove	Malta
Sparrowhawk	T15-007	*T. gallinae*	A/B	KC215387	**99%**	97%	Band-tailed pigeon	USA
Sparrowhawk	T17-066	*T. gallinae*	A/B	KC215387	100%	100%	Band-tailed pigeon	USA

Order+<br>Host species	Sample ID	*Trichomonas species*	Lineage	Accession number	Identity	Query cover	Host species	country
**Anseriformes**								
Egyptian goose	T17-079	*T. gallinae*	C/V/N	KX459498	100%	100%	Turtle dove	Spain
**Columbiformes**								
Collared dove	T15-032	*T. gallinae*	C/V/N	KX459497	100%	99%	Turtle dove	Spain
Feral Pigeon	T15-005	*T. gallinae*	C/V/N	KX459485	**99%**	99%	Turtle dove	Spain
Feral Pigeon	T15-025	*T. gallinae*	A/B	KX459444	100%	99%	Stock dove	Germany
Feral Pigeon	T15-038	*T. gallinae*	C/V/N	KX459498	**99%**	100%	Turtle dove	Spain
Feral Pigeon	T16-003	*T. gallinae*	C/V/N	KX459505	100%	100%	Turtle dove	Italy
Feral Pigeon	T16-005	*T. gallinae*	C/V/N	KX459498	100%	100%	Turtle dove	Spain
Feral Pigeon	T16-029	*T. gallinae*	C/V/N	KX459505	100%	100%	Turtle dove	Italy
Feral Pigeon	T16-069	*T. gallinae*	C/V/N	KX459505	100%	100%	Turtle dove	Italy
Feral Pigeon	T17-016	*T. gallinae*	A/B	KX459474	**99%**	100%	Woodpigeon	Germany
Feral Pigeon	T17-070	*T. gallinae*	C/V/N	KX459475	100%	100%	Woodpigeon	Germany
Feral Pigeon	T17-138	*T. gallinae*	C/V/N	KX459498	100%	100%	Turtle dove	Spain
Feral Pigeon	T17-156	*T. gallinae*	A/B	KX844991	100%	100%	Collared dove	Malta
Feral Pigeon	T17-160	*T. gallinae*	A/B	KC215387	100%	99%	Band-tailed pigeon	USA
Feral Pigeon	T17-162	*T. gallinae*	A/B	KX844991	100%	100%	Collared dove	Malta
Feral Pigeon	T17-167	*T. gallinae*	C/V/N	KX459475	100%	100%	Woodpigeon	Germany
Feral Pigeon	T17-169	*T. gallinae*	C/V/N	KX459499	100%	100%	Turtle dove	Spain
Stock dove	T17-124	*T. gallinae*	A/B	KX844991	100%	100%	Collared dove	Malta
Woodpigeon	T15-014	*T. gallinae*	C/V/N	KX459498	100%	100%	Turtle dove	Spain
Woodpigeon	T15-026	*T. gallinae*	C/V/N	KX459505	100%	99%	Turtle dove	Italy
Woodpigeon	T15-033	*T. gallinae*	C/V/N	KX459498	100%	99%	Turtle dove	Spain
Woodpigeon	T15-040	*T. gallinae*	C/V/N	KX459498	100%	100%	Turtle dove	Spain
Woodpigeon	T15-104	*T. gallinae*	C/V/N	KX459474	**99%**	100%	Woodpigeon	Germany
Woodpigeon	T16-040	*T. gallinae*	C/V/N	KX459505	100%	100%	Turtle dove	Italy

Order+<br>Host species	Sample ID	*Trichomonas species*	Lineage	Accession number	Identity	Query cover	Host species	country
Woodpigeon	T16-055	*T. gallinae*	C/V/N	KX459505	100%	100%	Turtle dove	Italy
Woodpigeon	T16-062	*T. gallinae*	A/B	KX844991	**99%**	100%	Collared dove	Malta
Woodpigeon	T16-163	*T. gallinae*	A/B	KX844991	100%	100%	Collared dove	Malta
Woodpigeon	T17-003	*T. gallinae*	C/V/N	KX459498	100%	100%	Turtle dove	Spain
Woodpigeon	T17-025	*T. gallinae*	C/V/N	KX459475	100%	100%	Woodpigeon	Germany
Woodpigeon	T17-073	*T. gallinae*	C/V/N	KX459475	100%	100%	Woodpigeon	Germany
Woodpigeon	T17-082	*T. gallinae*	C/V/N	KX459475	100%	100%	Woodpigeon	Germany
Woodpigeon	T17-090	*T. gallinae*	A/B	KX844991	100%	100%	Collared dove	Malta
Woodpigeon	T17-108	*T. gallinae*	C/V/N	KX459498	100%	100%	Turtle dove	Spain
Woodpigeon	T17-112	*T. gallinae*	C/V/N	KX459498	100%	100%	Turtle dove	Spain
Woodpigeon	T17-154	*T. gallinae*	C/V/N	KX459499	100%	100%	Turtle dove	Spain
**Falconiformes**								
Common kestrel	T15-015	*T. gallinae*	C/V/N	KX459475	**99%**	98%	Woodpigeon	Germany
Common kestrel	T16-057	*T. gallinae*	A/B	KX844991	100%	100%	Collared dove	Malta
Common kestrel	T16-076	*T. gallinae*	II	KX844984	100%	100%	Turtle dove	Malta
Common kestrel	T17-088	*T. gallinae*	C/V/N	KX459498	100%	100%	Turtle dove	Spain
Peregrine falcon	T17-013	*T. gallinae*	A/B	KX584000	100%	100%	Canary	Slovenia
**Passeriformes**								
Blackbird	T15-053	*T. gallinae*	C/V/N	KX459475	**99%**	99%	Woodpigeon	Germany
Blackbird	T15-055	*T. gallinae*	C/V/N	KX459475	100%	99%	Woodpigeon	Germany
Blackbird	T15-066	*T. gallinae*	A/B	KC215387	100%	99%	Band-tailed pigeon	USA
Blackbird	T15-077	*T. gallinae*	A/B	KC215387	**99%**	99%	Band-tailed pigeon	USA
Blackbird	T15-078	*T. gallinae*	C/V/N	KX459474	100%	100%	Woodpigeon	Germany
Blackbird	T15-081	*T. tenax*	-	KX459453	**98%**	99%	Stock dove	Germany
Chaffinch	T14-BF2	*T. tenax*	-	KX459511	100%	100%	Turtle dove	Italy

Order+<br>Host species	Sample ID	*Trichomonas species*	Lineage	Accession number	Identity	Query cover	Host species	country
Chaffinch	T15-086	*T. gallinae*	-	KC215387	**99%**	99%	Band-tailed pigeon	USA
Chaffinch	T17-157	*T. gallinae*	A/B	KX844991	100%	100%	Collared dove	Malta
European Robin	T15-064	*T. gallinae*	C/V/N	KX459498	100%	100%	Turtle dove	Spain
Greenfinch	T14-GF1	*T. gallinae*	A/B	KX584000	100%	97%	Canary	Slovenia
Greenfinch	T17-091	*T. gallinae*	A/B	KC215387	100%	99%	Band-tailed pigeon	USA
**Strigiformes**								
Barn owl	T15-019	*T. gallinae*	C/V/N	KX459498	**99%**	99%	Turtle dove	Spain
Barn owl	T15-102	*T. gallinae*	C/V/N	KX459498	100%	99%	Turtle dove	Spain
Eagle Owl	T15-006	*T. gallinae*	C/V/N	KX459474	100%	100%	Woodpigeon	Germany
Eagle Owl	T15-041	*T. gallinae*	C/V/N	KX459474	**99%**	100%	Woodpigeon	Germany
Eagle Owl	T16-075	*T. gallinae*	II	KX844987	**99%**	100%	Turtle dove	Malta
Long-eared owl	T15-028	*T. gallinae*	C/V/N	KX459475	**99%**	99%	Woodpigeon	Germany
Tawny Owl	T15-017	*T. gallinae*	C/V/N	KX459475	100%	100%	Woodpigeon	Germany
Tawny Owl	T15-024	*T. gallinae*	C/V/N	KX459474	100%	100%	Woodpigeon	Germany
