# Supplementary material for: Prevalence and genotyping of Trichomonas infections in wild birds in central Germany
Source: PLoS One. 2018 Aug 9;13(8):e0200798. doi: 10.1371/journal.pone.0200798 (PMC6084888; doi:10.1371/journal.pone.0200798)

Prevalence and genotyping of Trichomonas infections in wild birds in central Germany

Petra Quillfeldt, Yvonne R. Schumm, Carina Marek, Viktoria Mader, Dominik Fischer and Melanie Marx

Supplementary material

S1 Fig. Decline in greenfinch numbers in Hesse, Germany, according to the summer (https://www.nabu.de/tiere-und-pflanzen/aktionen-und-projekte/stunde-der-gartenvoegel/index.html) and winter garden bird counts (https://www.nabu.de/tiere-und-pflanzen/aktionen-und-projekte/stunde-der-wintervoegel/index.html) of the NABU (Nature and Biodiversity Conservation Union, Germany). Counts are carried out as citizen science projects in May and January, respectively.

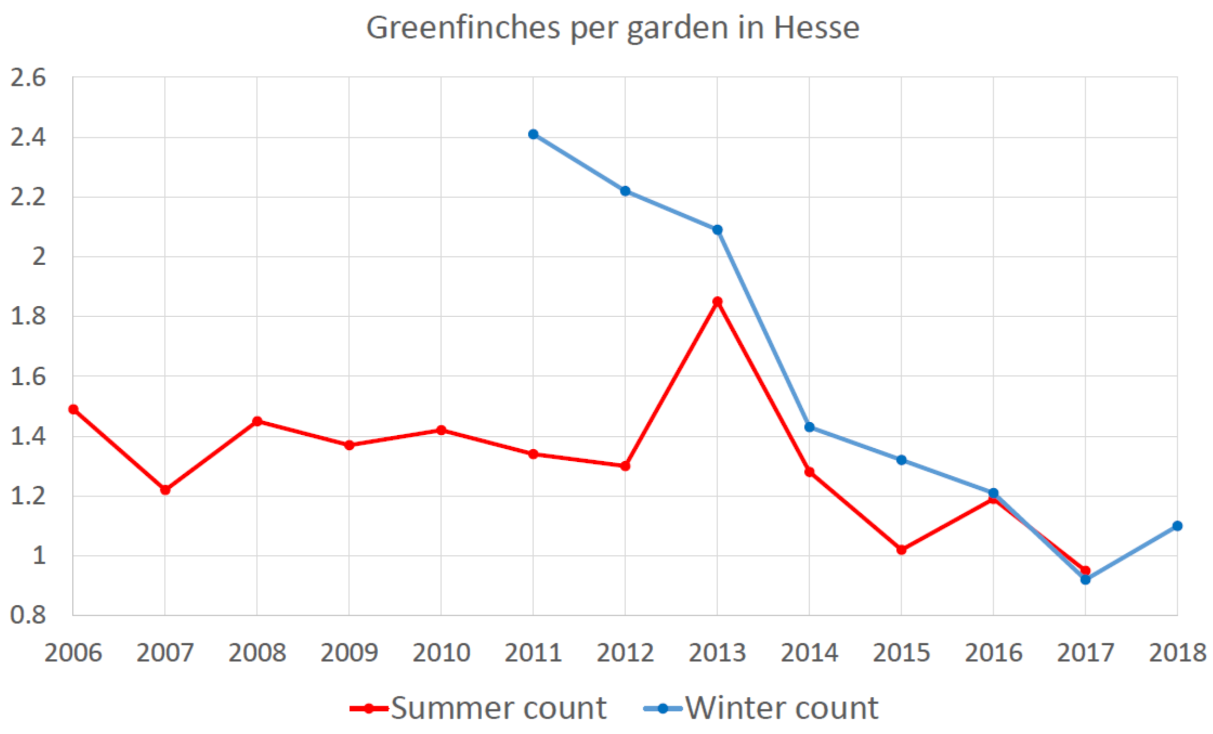

Supplement: S1 Fig — (PDF) [file pone.0200798.s002.pdf]
